# High preservation rates of the ascending branch of the lateral femoral circumflex artery during total hip arthroplasty through the direct anterior approach

**DOI:** 10.1002/jeo2.70066

**Published:** 2024-10-30

**Authors:** Christian Matar, Camille Vorimore, ReSurg, Sonia Ramos‐Pascual, Kinga Michalewska, Mo Saffarini, Frederic Laude

**Affiliations:** ^1^ Department of Orthopaedic Surgery Clinique du Sport Paris V, Ramsay Santé Paris France; ^2^ Department of Research ReSurg SA Nyon Switzerland

**Keywords:** a‐LFCA, ascending branch of the lateral femoral circumflex artery, circumflex arterio‐venous bundle, direct anterior approach, minimally invasive hip surgery, total hip arthroplasty

## Abstract

**Purpose:**

(i) To investigate the rate of preservation of the ascending branch of the lateral femoral circumflex artery (a‐LFCA) during total hip arthroplasty (THA) through a direct anterior approach (DAA), and (ii) to study factors that contribute to its successful preservation.

**Methods:**

All patients who underwent primary THA between 1 September 2023 and 29 February 2024 were reviewed. One‐hundred seventy‐two patients were included in the study, 91 females and 81 males, aged 63.2 ± 12.6, with a body mass index of 26.1 ± 4.6 kg/m^2^. THA was performed through a minimally invasive DAA in all patients, using either a standard vertical DAA or a Bikini incision. Patients were stratified by the preservation of the a‐LFCA (preserved, ligated/electrocauterized due to obstruction of the surgical field during dissection, electrocauterized due to bleeding during femoral broaching or stem insertion). Descriptive statistics were used to summarise the data. Logistic regression analyses were performed to explore possible factors associated with the preservation of a‐LFCA.

**Results:**

The a‐LFCA was preserved in 130 patients (75.6%), had to be ligated/electrocauterized due to obstruction of the surgical field during dissection in 31 patients (18.0%), and had to be electrocauterized due to bleeding during femoral broaching or stem insertion in 11 patients (6.4%). Multivariable logistic regression analyses revealed that preservation of a‐LFCA was significantly more likely in female patients (odds ratio [OR] = 2.22; 95% confidence interval [CI] = 1.1–4.6; *p* = 0.029), as well as in patients with younger age (OR = 0.97; 95% CI = 0.9–1.0; *p* = 0.036) and lower weight (OR = 0.97; 95% CI = 0.9–1.0; *p* = 0.005).

**Conclusions:**

Preservation of the a‐LFCA is possible in the majority of patients (75.6%) undergoing THA through minimally invasive DAA. Furthermore, preservation of the a‐LFCA is more likely in female patients, younger patients and patients with lower weight.

**Level of Evidence:**

Level IV.

**Abbreviations**
a‐LFCA: ascending branch of the lateral femoral circumflex artery
AO: acetabular offset
AP: anteroposterior
AVN: avascular necrosis
BMI: body mass index
CI: confidence intervals
DAA: direct anterior approach
DAG: directed acyclic graphs
FHEI: femoral head extrusion index
FO: femoral offset
GT‐ASIS: distance between the greater trochanter and anterior superior iliac spine
IFD: ischiofemoral distance
IIR: ilio‐ischial ratio
LCEA: lateral centre edge angle
NSA: femoral neck‐shaft angle
OR: odds ratios
TFLM: tensor fasciae latae muscle
THA: total hip arthroplasty

## INTRODUCTION

The direct anterior approach (DAA) in total hip arthroplasty (THA) has witnessed a consistent rise in popularity over the past two decades [3, 4, 22, 23, 24]. The DAA preserves the muscles and soft tissues around the hip, thus resulting in good early postoperative outcomes [1, 9, 17, 19, 20]. Furthermore, the DAA may result in a lower risk of complications compared to other surgical approaches, although it has a steeper learning curve [18].

The surgical technique of the original DAA and its variants involves the ligation or electrocauterization of the ascending branch of the lateral femoral circumflex artery (a‐LFCA) to facilitate safe surgical access to the hip joint [22]. Several clinical and cadaveric studies have shown that the a‐LFCA is the main blood supply of the tensor fasciae latae muscle (TFLM) [6, 10, 11, 21]. Therefore, ligating or electrocauterizing the a‐LFCA during DAA could result in reduced perfusion of the TFLM [10], which could manifest in transient ischaemic pain or claudication.

Although preserving the a‐LFCA during THA may provide benefits to the patient, no prior studies have evaluated the feasibility of preserving it. Over the last year, the senior author systematically attempted to preserve the a‐LFCA during THA through the DAA. The purpose of the present study was (i) to investigate the rate of preservation of the a‐LFCA when performing THA through a minimally invasive DAA and (ii) to study factors that contribute to its successful preservation.

## MATERIALS AND METHODS

The authors retrospectively reviewed all patients (*n* = 178) who underwent primary THA between 1 September 2023 and 29 February 2024, operated on by one senior surgeon with more than 30 years of experience (F. L.). The surgeon systematically performed the DAA for all primary THAs. The exclusion criteria for the present study were (i) patients with severely deformed hips due to sequelae of Perthes disease (*n* = 3), (ii) patients with Crowe 3 and 4 dysplastic hips (*n* = 2) or (iii) patients operated for a femoral neck fracture (*n* = 1).

The cohort comprised 172 patients, 91 females and 81 males, aged 63.2 ± 12.6 (range, 27–97) with a body mass index (BMI) of 26.1 ± 4.6 kg/m^2^ (range, 18–50 kg/m^2^) (Table 1). Indications for THA included primary osteoarthritis (*n* = 145), secondary osteoarthritis (*n* = 26) and avascular necrosis (*n* = 1). This study was approved by the institutional review board of ‘GCS Ramsay Santé pour l'Enseignement et la Recherche’ (#IRB: COS‐RGDS‐2024‐02‐004‐LAUDE‐F). Informed consent was obtained from all individual participants included in the study.

**Table 1:** Preoperative characteristics for the cohort, stratified by preservation of a‐LFCA (*n* = 172).

	Group A			Group B				Group C				
	Preserved a‐LFCA (*n* = 130)			Ligated/electrocauterized a‐LFCA due to obstruction of the surgical field during dissection (*n* = 31)				Electrocauterized a‐LFCA due to bleeding during femoral broaching or stem insertion (*n* = 11)				
	Mean ± SD		(Range)	Mean ± SD		(Range)		Mean ± SD		(Range)		*p* Value
	*n* (%)			*n* (%)				*n* (%)				
Age (years)	62.1 ± 12.3		(26.5–97.2)	65.9 ± 13.7		(42.6–90.4)		69.2 ± 11.3		(51.2–89.5)		0.110
Weight (kg)	74.2 ± 14.3		(48.0–130.0)	85.9 ± 19.7		(58.0–140.0)		72.9 ± 16.8		(50.0–102.0)		**0.008**
Height (cm)	170.2 ± 8.0		(150.0–188.0)	172.7 ± 9.6		(157.0–197.0)		168.9 ± 11.7		(156.0–190.0)		0.298
BMI (kg/m^2^)	25.5 ± 4.1		(18.0–43.9)	28.7 ± 5.7		(21.3–49.6)		25.3 ± 3.6		(19.7–31.1)		**0.008**
Sex												**0.039**
Female	75 (57.7%)			10 (32.3%)				6 (54.5%)				
Male	55 (42.3%)			21 (67.7%)				5 (45.5%)				
Indication												0.057
Primary OA	110 (84.6%)			27 (87.1%)				8 (72.7%)				
OA secondary to dysplasia	19 (14.6%)			2 (6.5%)				2 (18.2%)				
OA secondary to Perthes disease	0 (0.0%)			1 (3.2%)				1 (9.1%)				
OA secondary to retroversion	1 (0.8%)			0 (0.0%)				0 (0.0%)				
Avascular necrosis	0 (0.0%)			1 (3.2%)				0 (0.0%)				
Kellgren–Lawrence grade												0.843
Not appropriate^a^	0 (0.0%)			1 (3.2%)				0 (0.0%)				
2	3 (2.3%)			1 (3.2%)				0 (0.0%)				
3	42 (32.3%)			7 (22.6%)				4 (36.4%)				
4	85 (65.4%)			22 (71.0%)				7 (63.6%)				
Dorr type												0.755
A	13 (10.0%)			3 (9.7%)				0 (0.0%)				
B	115 (88.5%)			28 (90.3%)				11 (100.0%)				
C	2 (1.5%)			0 (0.0%)				0 (0.0%)				
Acetabular offset (mm)	34.5 ± 4.8		(25.0–48.0)	36.1 ± 4.6		(30.0–47.0)		33.1 ± 5.8		(26.0–42.0)		0.250
Femoral offset (mm)	52.1 ± 5.3		(42.0–70.0)	54.1 ± 7.1		(43.0–72.0)		51.3 ± 8.2		(42.0–64.0)		0.506
Neck shaft angle (°)	133.3 ± 6.9		(112.0–150.0)	133.2 ± 6.0		(123.0–148.0)		134.7 ± 6.2		(125.0–143.0)		0.642
LCEA (°)	29.8 ± 7.9		(10.0–49.0)	30.4 ± 6.3		(18.0–45.0)		31.0 ± 8.1		(19.0–47.0)		0.984
GT‐ASIS (mm)	106.5 ± 10.9		(77.0–139.0)	108.0 ± 10.5		(88.0–130.0)		106.2 ± 8.6		(96.0–121.0)		0.812
IFD (mm)	25.4 ± 8.8		(6.0–47.0)	24.7 ± 6.9		(13.0–45.0)		23.0 ± 8.9		(12.0–37.0)		0.601
IIR	2.9 ± 0.5		(2.0–4.4)	2.9 ± 0.4		(1.9–3.6)		2.9 ± 0.6		(1.9–3.6)		0.770
FHEI	0.2 ± 0.1		(0.0–0.5)	0.2 ± 0.1		(0.0–0.3)		0.2 ± 0.1		(0.1–0.3)		0.709

*Note*: *p* Values in bold are statistically significant.

Abbreviations: a‐LFCA, ascending lateral circumflex artery; BMI, body mass index; FHEI, femoral head extrusion index; GT‐ASIS, distance between greater trochanter and anterior superior iliac spine; IFD, ischio‐femoral distance; IIR, ilio‐ischial ratio; LCEA, lateral centre edge angle; OA, osteoarthritis; SD, standard deviation.

^a^ Patient with avascular necrosis did not have a Kellgren‐Lawrence grade.

### Surgical technique

**Figure 1:**
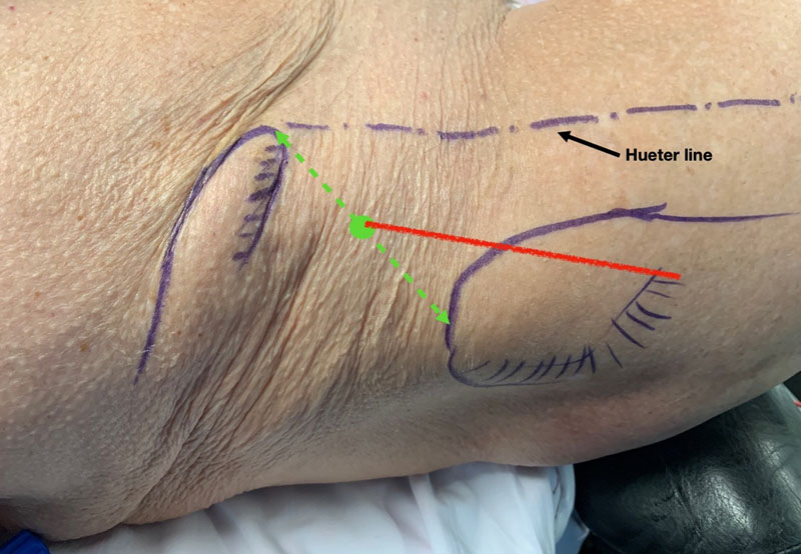
The standard vertical incision (in red) started between the antero‐superior iliac spine and the summit of the greater trochanter and diverged slightly from the Hueter line laterally.

**Figure 2:**
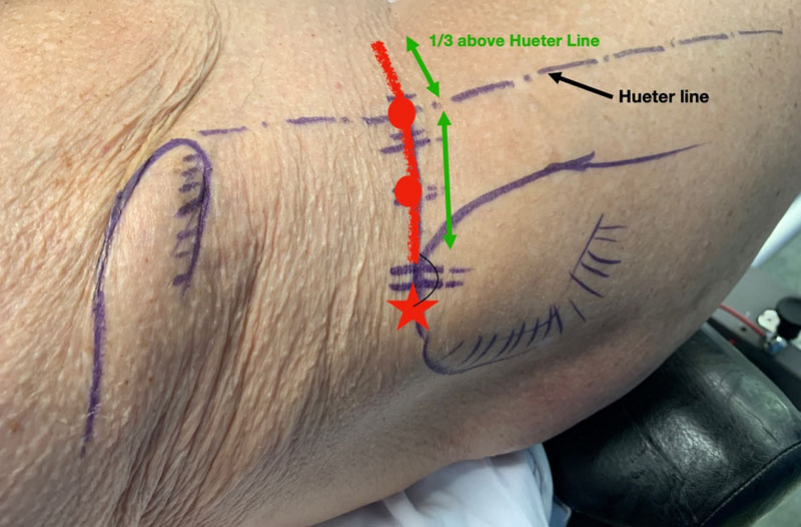
The ‘Bikini’ incision (in red) was perpendicular to the Hueter line, with the lateral part of the incision finishing at the summit of the greater trochanter.

**Figure 3:**
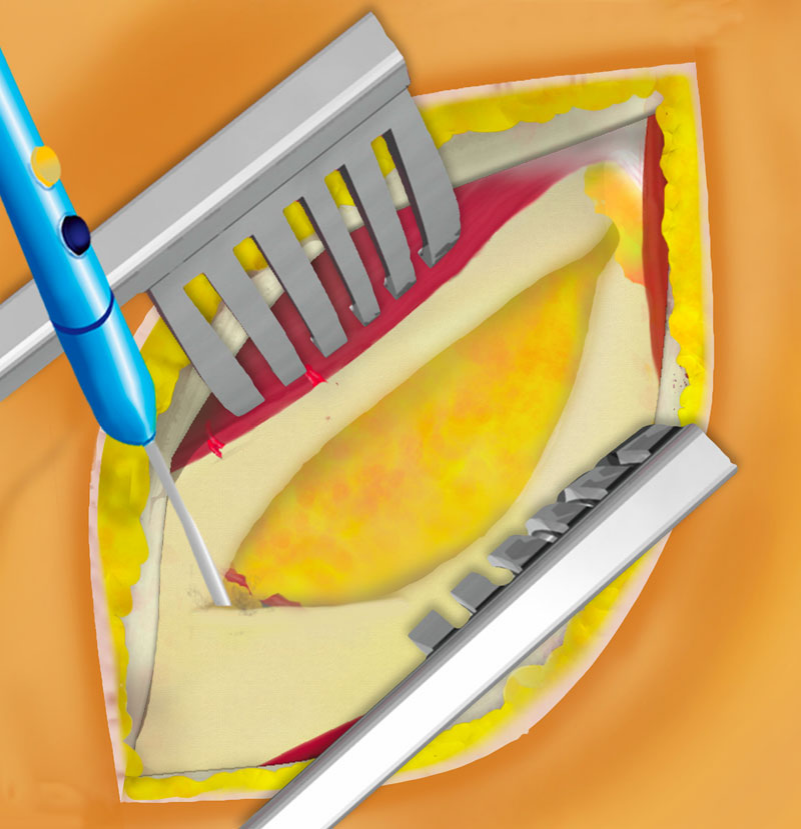
A Beckmann retractor was inserted to hold the tensor fasciae latae muscle and sartorius muscle in place. Dissection of the innominate fascia was then limited distally to the level of the circumflex arterio‐venous bundle.

**Figure 4:**
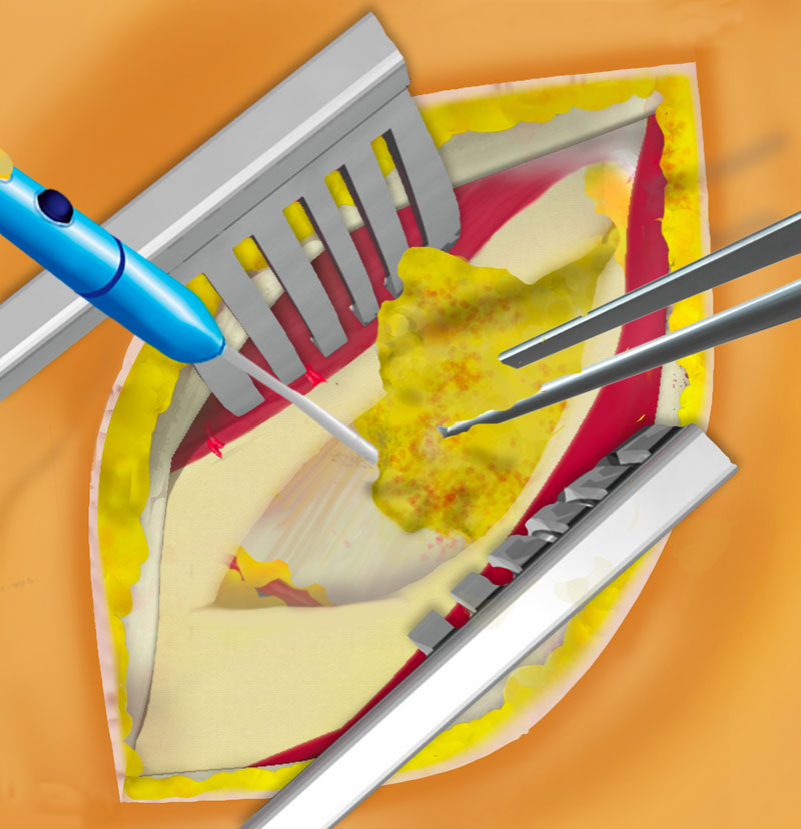
Precapsular fatty tissue was then excised without damaging the vascular pedicle.

**Figure 5:**
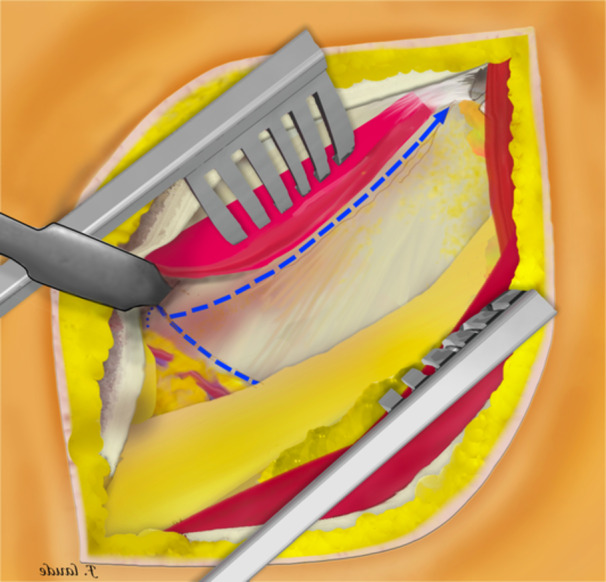
An L‐shape capsulotomy was performed, with the vascular pedicle protected in its sheath, during left‐side total hip arthroplasty through direct anterior approach.

**Figure 6:**
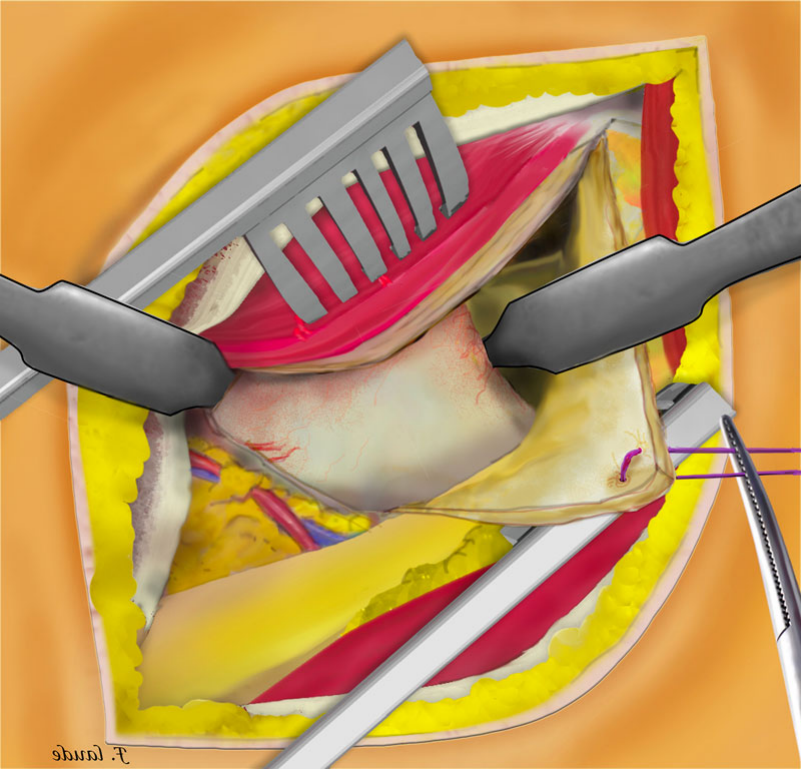
The Hohmann retractors were inserted medially into the capsule, protecting the circumflex arterio‐venous bundle.

This surgical technique is based on a mini‐open DAA technique, previously described 15 years ago for the treatment of femoroacetabular impingement [14]. The patient was positioned supine on the operating table, with the use of a leg positioner (AMIS mobile leg positioner, Medacta) [5, 8, 13]. A standard vertical DAA incision was performed in males [5, 13], and a skin crease ‘Bikini’ incision [15, 16] was performed in females for cosmetic reasons, both with a length of 5–8 cm. The standard vertical incision started between the antero‐superior iliac spine (ASIS) and the summit of the greater trochanter and diverged slightly from the Hueter line laterally (Figure 1). The ‘Bikini’ incision was perpendicular to the Hueter line, with the lateral part of the incision finishing at the summit of the greater trochanter (Figure 2). Of note, on four females, the standard vertical DAA incision was performed since an incision extension was anticipated for acetabular augmentation (Table 2). Additionally, on five very obese males, the ‘Bikini’ incision was performed in the anatomical skin crease to avoid the widening of the scar and potential complications. A subcutaneous dissection was then performed longitudinally on all patients. The aponeurosis of the TFLM was identified and incised. The TFLM was then bluntly mobilised off the fascia laterally, protecting the lateral femoral cutaneous nerve. A blunt dissection was then carried out between the TFLM and the sartorius and rectus femoris muscles. During this step, a Beckmann retractor was inserted to hold the muscles in place [5]. The fascia innominata was then carefully incised without exposing the ascending branch of the circumflex arteriovenous bundle, which was identified and taken as the landmark to limit the dissection distally (Figure 3). Then the innominate fascia was visualised and dissected between the rectus femoris and the gluteal muscles, no further distally than the circumflex arterio‐venous bundle, when possible. In such cases, the a‐LFCA was left intact in its sheath, protected by the surrounding fatty tissue. The precapsular fatty tissue was then excised (Figure 4). Hohmann retractors were inserted medially into the capsule, protecting the circumflex arterio‐venous bundle, while performing an L‐shaped capsulotomy (Figures 5 and 6). If it was not possible to keep the a‐LFCA intact in its sheath, it was ligated or electrocauterized, before performing the L‐shaped capsulotomy. A femoral neck osteotomy was then performed using an oscillating saw, and the femoral head was removed with a corkscrew. The osteotomy was performed from the upper part of the pretrochanteric tubercle toward the Hohmann retractor, which was placed on the medial neck just above the lesser trochanter. The circumflex arterio‐venous bundle usually passed below the pretrochanteric tubercle, and thus was not generally damaged by the osteotomy, as the Hohmann retractor was protecting the medial part of the bundle. A modified Charnley‐frame retractor was inserted on the lateral and medial capsular flaps, preserving the TFLM and the rectus femoris [26]. The acetabulum was gradually reamed to the appropriate size, and the desired depth, version, and inclination, then the acetabular components were implanted.

**Table 2:** Surgical data for the cohort, stratified by preservation of a‐LFCA (*n* = 172).

	Group A				Group B				Group C				
	Preserved a‐LFCA (*n* = 130)				Ligated/electrocauterized a‐LFCA due to obstruction of surgical field during dissection (*n* = 31)				Electrocauterized a‐LFCA due to bleeding during femoral broaching or stem insertion (*n* = 11)				
	Mean ± SD		(Range)		Mean ± SD		(Range)		Mean ± SD		(Range)		*p* Value
	*n* (%)				*n* (%)				*n* (%)				
Surgery time (min)	39.8 ± 3.6		(35–65)		41.4 ± 5.1		(37–60)		42.2 ± 6.6		(37–59)		0.119
Operated side													0.536
Left	54 (41.5%)				16 (51.6%)				4 (36.4%)				
Right	76 (58.5%)				15 (48.4%)				7 (63.6%)				
Incision type													**0.022**
Bikini	77 (59.2%)				10 (32.3%)				5 (45.5%)				
Standard vertical DAA	53 (40.8%)				21 (67.7%)				6 (54.5%)				
Stem type													0.700
Amistem	111 (85.4%)				28 (90.3%)				10 (90.9%)				
SMS	19 (14.6%)				3 (9.7%)				1 (9.1%)				
Collared stem													0.248
Yes	40 (30.8%)				9 (29.0%)				6 (54.5%)				
No	90 (69.2%)				22 (71.0%)				5 (45.5%)				
Cup diameter (mm)													0.449
44–48	39 (30.0%)				7 (22.6%)				4 (36.4%)				
50–54	75 (57.7%)				18 (58.1%)				4 (36.4%)				
56–62	16 (12.3%)				6 (19.4%)				3 (27.3%)				
Head diameter (mm)													0.108
28	10 (7.7%)				6 (19.4%)				1 (9.1%)				
32	103 (79.2%)				18 (58.1%)				7 (63.6%)				
36	17 (13.1%)				7 (22.6%)				3 (27.3%)				
Acetabular graft													0.179
Yes	6 (4.6%)				2 (6.5%)				2 (18.2%)				
No	124 (95.4%)				29 (93.5%)				9 (81.8%)				

*Note*: *p* Values in bold are statistically significant.

Abbreviations: a‐LFCA, ascending lateral circumflex artery; DAA, direct anterior approach; SD, standard deviation.

**Figure 7:**
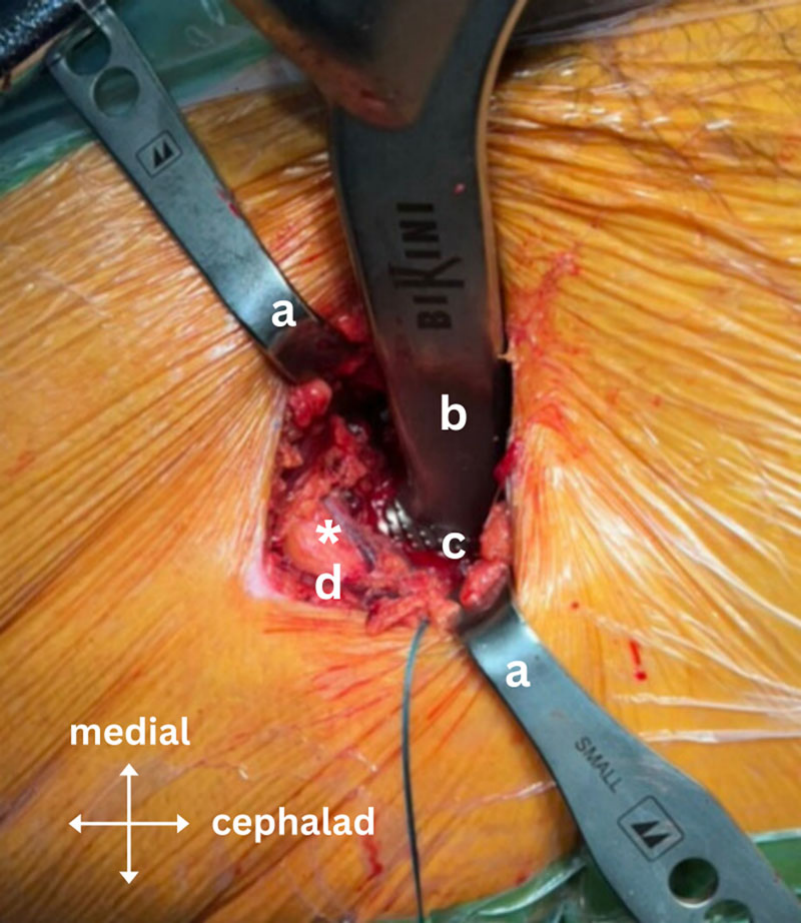
Intraoperative image showing the intact circumflex arterio‐venous bundle during femoral broaching using the Bikini Broach Handle (Medacta) and the modified Charnley‐frame retractor inserted on the lateral and medial capsular flaps, preserving the tensor fasciae latae muscle (TFLM) and the rectus femoris; where ‘a’ is the modified Charnley‐frame retractor, ‘b’ is the Bikini Broach Handle, ‘c’ is the femoral broach, ‘d’ is the TFLM and ‘*’ is the circumflex arterio‐venous bundle in its sheath surrounded by fatty tissue.

**Figure 8:**
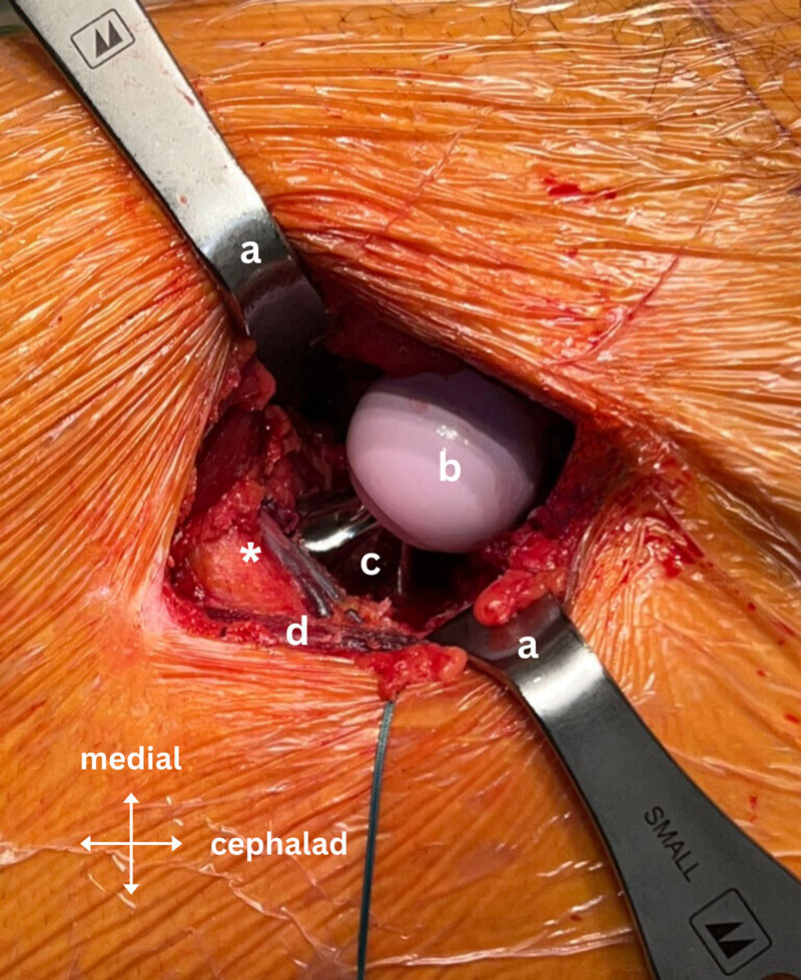
Intraoperative image showing the intact circumflex arterio‐venous bundle after implantation of the stem and head; where ‘a’ is the modified Charnley‐frame retractor, ‘b’ is the ceramic femoral head, ‘c’ is the femoral stem, ‘d’ is the tensor fasciae latae muscle and ‘*’ is the circumflex arterio‐venous bundle in its sheath surrounded by fatty tissue.

The leg was then positioned in external rotation and hyperextension to expose the femoral canal. In most cases, there was no need for capsular or soft tissue release, due to the use of an offset broach handle (Bikini Broach Handle, Medacta) and the external rotation achieved by the operating table. The canal was then broached sequentially up to the appropriate size, according to preoperative templating, until the fitted femoral stem was stable. During the study period, the surgeon's preferred stem for primary THA was a shortened double‐tapered uncemented collarless stem (Amistem‐P); however, a metaphyseal‐filling short stem (SMS) was used for patients with Dorr A femurs. Care was taken to protect the circumflex arterio‐venous bundle during femoral broaching and stem impaction by using a simple Army‐Navy retractor (Figure 7). The femoral head was then impacted onto the femoral stem, and the prosthesis was reduced (Figure 8). The surgical site was inspected, and if bleeding from the a‐LFCA was detected, electrocauterization was performed. Closure of the surgical site was performed in layers. Surgical notes documented whether the a‐LFCA had to be ligated or electrocauterized, as well as any intraoperative complications.

### Preoperative radiographic assessment

Standard anteroposterior pelvic radiographs were assessed to evaluate the acetabular offset, femoral offset, the femoral neck‐shaft angle, the lateral centre edge angle, the distance between the greater trochanter and anterior superior iliac spine (GT‐ASIS), the ischiofemoral distance (IFD), the ilio‐ischial ratio (IIR) and the femoral head extrusion index (FHEI) (Table 1).

### Statistical analysis

Descriptive statistics were used to summarise the data, which was stratified by the preservation of the a‐LFCA (preserved, ligated/electrocauterized due to obstruction of the surgical field during dissection, electrocauterized due to bleeding during femoral broaching or stem insertion). Since one of the groups (electrocauterized due to bleeding during femoral broaching or stem insertion, *n* = 11) had <30 patients, data was considered non‐normally distributed [2, 12]. Thus, comparisons between groups were performed using Kruskal–Wallis tests for continuous variables and using Chi‐squared tests for categorical variables. Univariable logistic regression analyses were performed to explore possible associations between the preservation of a‐LFCA (binary outcome, yes/no) and all independent variables. Associations were presented as odds ratios (OR) with their corresponding 95% confidence intervals (CI) and *p* values. Multivariable logistic regression analyses were performed after the selection of pertinent variables using directed acyclic graphs (DAG) (Supporting Information S1: Appendix 1). Subgroups with <5 patients were not included in regression analyses as the sample size was considered insufficient. Of note, incision type was taken as a confounding factor for sex, since the location of the a‐LFCA may be dependent on sex [7, 25]. Statistical analyses were conducted using R, version 4.3.1 (R Foundation for Statistical Computing). *p* < 0.05 were considered statistically significant.

## RESULTS

Of the 172 patients in the present study, the surgeon was able to preserve the a‐LFCA in 130 (75.6%; Group A), but had to ligate/electrocauterize the a‐LFCA due to obstruction of the surgical field during dissection in 31 (18.0%; Group B), and had to electrocauterize the a‐LFCA due to bleeding during femoral broaching or stem insertion in 11 (6.4%; Group C). There were no intraoperative complications, such as excessive bleeding.

Group B had a significantly greater weight (85.9 ± 19.7 kg) and BMI (28.7 ± 5.7 kg/m^2^) compared to Groups A (74.2 ± 14.3 kg; 25.5 ± 4.1 kg/m^2^) and C (72.9 ± 16.8 kg, *p* = 0.008; 25.3 ± 3.6 kg/m^2^, *p *= 0.008) (Table 1). Furthermore, Group B had a significantly lower proportion of females (32.3%) and proportion of patients with a ‘Bikini’ incision (32.3%) compared to Groups A (57.7%; 59.2%) and C (54.5%, *p* = 0.039; 45.5%, *p* = 0.022).

Univariable logistic regression analyses revealed that preservation of the a‐LFCA was significantly more likely in female patients (OR = 2.22; 95% CI = 1.1–4.6; *p *= 0.029) and patients with ‘Bikini’ incision (OR = 2.62; 95% CI = 1.29–5.49; *p *= 0.009); furthermore the preservation of the a‐LFCA was associated with younger age (OR = 0.97; 95% CI = 0.9–1.0; *p *= 0.036), lower weight (OR = 0.97; 95% CI = 0.9–1.0; *p *= 0.005) and lower BMI (OR = 0.90; 95% CI = 0.8–1.0; *p *= 0.009) (Table 3). Of note, sex and incision type were highly correlated, with 87 of 91 females and five of 81 males getting a ‘Bikini’ incision. Additionally, multivariable logistic regression analyses confirmed that preservation of the a‐LFCA was significantly more likely in female patients (OR = 2.22; 95% CI = 1.1–4.6; *p* = 0.029), as well as in patients with younger age (OR = 0.97; 95% CI = 0.9–1.0; *p* = 0.036) and lower weight (OR = 0.97; 95% CI = 0.9–1.0; *p* = 0.005). None of the morphologic hip parameters were associated with preservation of the a‐LFCA.

**Table 3:** Logistic regression analyses to identify factors associated with the preservation of a‐LFCA (*n* = 172).

	Univariable				Multivariable			
	OR	(95% CI)		*p* Value	OR	(95% CI)		*p* Value
Age	0.97	(0.94–1.00)		**0.036**	0.97	(0.94–1.00)		**0.036**
Weight (kg)	0.97	(0.95–0.99)		**0.005**	0.97	(0.95–0.99)		**0.005**
Height (cm)	0.98	(0.94–1.02)		0.314	1.02	(0.97–1.09)		0.435
BMI (kg/m^2^)	0.90	(0.83–0.97)		**0.009**	1.72	(0.81–3.80)		0.162
Female sex	2.22	(1.10–4.60)		**0.029**	2.22	(1.10–4.60)		**0.029**
Indication								
Primary OA	REF				REF			
OA secondary to dysplasia	1.51	(0.53–5.47)		0.479	3.56	(0.67–28.89)		0.171
OA secondary to Perthes disease^a^								
OA secondary to retroversion^a^								
Avascular necrosis^a^								
Kellgren‐Lawrence grade								
Not appropriate^a^								
2^a^								
3	1.32	(0.61–2.99)		0.491	1.11	(0.50–2.57)		0.810
4	REF				REF			
Dorr type								
A	1.47	(0.45–6.65)		0.564	1.98	(0.55–9.46)		0.332
B	REF				REF			
C^a^								
Acetabular offset (mm)	0.97	(0.89–1.05)		0.413	1.00	(0.92–1.10)		0.918
Femoral offset (mm)	0.96	(0.90–1.03)		0.276	0.95	(0.88–1.03)		0.233
Neck shaft angle (°)	0.99	(0.94–1.05)		0.797	0.99	(0.94–1.05)		0.797
LCEA (°)	0.99	(0.94–1.04)		0.603	0.99	(0.94–1.04)		0.603
GT‐ASIS (mm)	0.99	(0.95–1.03)		0.630	1.00	(0.95–1.04)		0.836
IFD (mm)	1.02	(0.97–1.07)		0.498	1.06	(1.00–1.13)		0.074
IIR	1.03	(0.48–2.25)		0.941	2.27	(0.85–6.62)		0.116
FHEI	0.31	(0.00–24.30)		0.596	0.07	(0.00–17.83)		0.358
Left operated side	0.78	(0.39–1.58)		0.489	0.78	(0.39–1.58)		0.489
Bikini incision	2.62	(1.29–5.49)		**0.009**	3.48	(0.71–20.18)		0.137
SMS stem	1.63	(0.57–5.87)		0.403	1.59	(0.44–7.28)		0.505
Collared stem	0.80	(0.39–1.69)		0.551	0.77	(0.37–1.66)		0.501
Cup diameter								
44–48	1.04	(0.46–2.43)		0.925	0.80	(0.80–2.84)		0.732
50–54	REF				REF			
56–62	0.52	(0.20–1.38)		0.177	0.52	(0.15–1.77)		0.294

*Note*: *p* Values in bold are statistically significant.

Abbreviations: a‐LFCA, ascending lateral circumflex artery; BMI, body mass index; CI, confidence intervals; FHEI, femoral head extrusion index; GT‐ASIS, distance between the greater trochanter and anterior superior iliac spine; IFD, ischio‐femoral distance; IIR, ilio‐ischial ratio; LCEA lateral centre edge angle; OA, osteoarthritis; OR, odds ratio.

^a^ Less than five patients in subgroup: insufficient for regression analysis.

## DISCUSSION

The most important finding of the present study is that the rate of preservation of the a‐LFCA when performing THA through a minimally invasive DAA is 75.6%. Furthermore, the present study found that factors that may contribute to the preservation of the a‐LFCA are female sex (OR = 2.22; 95% CI = 1.1–4.6; *p* = 0.029), young age (OR = 0.97; 95% CI = 0.9–1.0; *p* = 0.036), and low weight (OR = 0.97; 95% CI = 0.9–1.0; *p* = 0.005). Experienced surgeons should aim to preserve the a‐LFCA during THA through DAA, as this could prevent reduced perfusion of the TFLM.

The present study showed that preservation of the a‐LFCA was possible in 75.6% of cases. However, 18.0% of cases had to be ligated/electrocauterized due to obstruction of the surgical field during dissection, and 6.4% of cases had to be electrocauterized due to bleeding during femoral broaching or stem insertion. Interestingly, the authors noticed that bleeding from a torn a‐LFCA occurred during femoral preparation (Group C) only in cases where the a‐LFCA was located close to the femur (<1 cm in the axial plane), due to increased tension on the a‐LFCA during external rotation of the leg. Remarkably, there were no postoperative hematomas or wound infections. It was not possible to compare the rate of preservation of the a‐LFCA during DAA in the present study to that in the literature, as no published study reported this.

The present study found that preservation of the a‐LFCA was significantly more likely in female patients (OR = 2.22; 95% CI = 1.1–4.6; *p* = 0.029), as well as in patients with younger age (OR = 0.97; 95% CI = 0.9–1.0; *p* = 0.036) and lower weight (OR = 0.97; 95% CI = 0.9–1.0; *p* = 0.005). It is important to note, however, that sex and incision type were highly correlated, with 87 of 91 females and five of 81 males getting a ‘Bikini’ incision. Therefore, it is not possible to conclude from the data in the present study if preservation of the a‐LFCA was more likely in female patients because of their anatomy or because they were operated on with a ‘Bikini’ incision. However, the only two studies [7, 25] in the literature that describe the anatomic position of the a‐LFCA found that there may be differences in position across sexes. Harper et al. [7] measured the ratio ‘distance from the lesser trochanter to the LFCA (LT) / total distance from the lesser trochanter to the greater trochanter (TD)’ in 108 patients undergoing THA through DAA and found a significant difference between females and males on fluoroscopic images (0.567 ± 0.158 vs. 0.638 ± 0.147; *p* = 0.017). Furthermore, Totlis et al. [25] dissected the hips of 23 human cadavers and found a significant difference between females and males in the vertical distance from the ASIS to the LFCA (99.2 ± 14.9 vs. 120.0 ± 13.7; *p* = 0.008), but no significant difference in the horizontal distance from the ASIS to the LFCA (66.6 ± 11.9 vs. 64.0 ± 19.6; *p *= 0.757). Interestingly, regression analyses in the present study revealed that preservation of the a‐LFCA is not associated with either the type of stem (*p* = 0.133) or the cup diameter (*p* ≥ 0.489).

The a‐LFCA is located one‐ to two‐thirds between the lesser and greater trochanters (10–13 cm distal to the anterior superior iliac spine) along the anatomic axis of the femur [7, 25], and thus spanning across the surgical field during THA through DAA. For this reason, the pioneer of the DAA routinely ligated/electrocauterized the a‐LFCA during this surgical approach. However, the recent literature has shown that the a‐LFCA is the main vascular supply to the TFLM, as well as an important contributor to articular and periarticular hip vascularisation [6, 10, 11, 21]. In addition, the transverse branch of the a‐LFCA contributes to the vascularisation of the greater trochanter [21]. Future studies should evaluate the effect of preserving the a‐LFCA during THA on functional outcomes. The present study describes a modification of THA through DAA that preserves the a‐LFCA. The surgical steps that contribute to sparing the a‐LFCA are limiting soft tissue release and maintaining the vascular pedicle within its sheath. This increases tissue extensibility during controlled external rotation and extension of the leg for femoral preparation. Furthermore, no posterior release was performed, and the femur was not elevated during any time of the operation, thus reducing the risk of tearing the a‐LFCA. Additionally, the combined use of the modified Charnley‐frame retractor, the optimized broach handle, and the leg holder facilitated controlled and precise external rotation during extension while ensuring optimal visualization of the vascular pedicle. We believe that this modified approach may be advantageous over other minimally invasive approaches, as no further retraction is exerted on the TFLM (since the traction is exerted on the capsule), thus reducing the risk of compression injuries.

Patients in Group B had their a‐LFCA either ligated or electrocauterized depending on surgeon preference, as a recent study by Zhao et al. [27] showed no significant differences between ligation and electrocauterization of the a‐LFCA in terms of blood loss, postoperative transfusions, postoperative hematomas or re‐bleeding. Furthermore, in cases where the a‐LFCA had to be ligated or electrocauterized, this was done as far as possible from the entry point to the TFLM to mitigate the risk of additional injury to the superior gluteal nerve, thus avoiding potential denervation [6].

Further imaging studies should investigate the effect of ligating/electrocauterizing the a‐LFCA on fatty infiltration or atrophy of the TFLM, and reduction in its cross‐sectional area, taking into consideration the pre‐existing fatty muscle degeneration in patients before surgery [17]. Furthermore, additional imaging and clinical studies on patients who undergo THA through DAA with preservation of the a‐LFCA are necessary to evaluate the soft tissues of the anterior region of the thigh, as well as the clinical and functional outcomes in the short to mid‐term.

The present retrospective study has a number of limitations. First, all surgeries were performed by a senior surgeon who is very experienced with the DAA. Therefore, the results may not be generalizable to other less experienced surgeons in this technique. Second, most female patients had a ‘Bikini’ incision, while most male patients had a standard vertical DAA incision. Therefore, we cannot conclude if incision type or sex independently influences the preservation of the a‐LFCA. Third, the anatomical location of the a‐LFCA was not recorded, which could have helped better understand why it was not possible to preserve the vessel in some cases. In addition, in cases where the vessel was preserved, its functional integrity and continued perfusion after surgery were not checked, because this was not routine clinical practice. However, future studies could confirm that the a‐LFCA remained intact by performing a Doppler ultrasound. Furthermore, no outcomes were collected, and, therefore, it is not possible to study the effect of preserving the a‐LFCA on patient‐reported outcomes.

## CONCLUSIONS

Preservation of the a‐LFCA is possible in the majority of patients (75.6%) undergoing THA through minimally invasive DAA. Furthermore, preservation of the a‐LFCA is more likely in female patients, younger patients, and patients with lower weight. Experienced surgeons should aim to preserve the a‐LFCA during THA through DAA, as this could prevent reduced perfusion of the TFLM.

## AUTHOR CONTRIBUTIONS

**Christian Matar**: Conceptualisation; data curation; methodology; writing—review and editing. **Camille Vorimore**: Conceptualisation; data curation; methodology; writing—review and editing. **Sonia Ramos‐Pascual**: Formal analysis; software; visualization; writing—original draft. **Kinga Michalewska**: Formal analysis; software; visualization; writing—original draft. **Mo Saffarini**: Formal analysis; software; visualization; writing—review and editing. **Frederic Laude**: Conceptualisation; data curation; methodology; project administration; resources; writing—review and editing.

## CONFLICTS OF INTEREST STATEMENT

F. L. declares royalties from Medacta. Remaining authors declare no conflict of interest.

## ETHICS STATEMENT

All patients provided informed consent for the use of their data for research and publications. The present work was completed after being approved by an institutional review board (COS‐RGDS‐2024‐02‐004‐LAUDE‐F).

## Supporting information

Supporting Information.

## Data Availability

The database used during the current study is available from the corresponding author upon reasonable request.

## References

[1] Bremer, A.K. , Kalberer, F. , Pfirrmann, C.W.A. & Dora, C. (2011) Soft‐tissue changes in hip abductor muscles and tendons after total hip replacement: comparison between the direct anterior and the transgluteal approaches. The Journal of Bone and Joint Surgery. British Volume, 93(7), 886–889. Available from: 10.1302/0301-620X.93B7.25058 21705558

[2] Chang, H.‐J. , Huang, K.‐C. & Wu, C.‐H. (2006) Determination of sample size in using central limit theorem for Weibull distribution. International Journal of Information and Management Sciences, 17, 31–46.

[3] Chang, J.S. , Kang, M.W. , Lee, D.H. , Kim, J.W. & Kim, C.‐H. (2023) Comparing the anterior‐based muscle‐sparing approach with the direct anterior approach in hip arthroplasty: a systematic review and pairwise meta‐analysis. Medicina, 59(8), 1390. Available from: 10.3390/medicina59081390 37629680 PMC10456498

[4] Galakatos, G.R. (2018) Direct anterior total hip arthroplasty. Missouri Medicine, 115(6), 537–541. 30643349 PMC6312152

[5] Gollwitzer, H. (2018) Die minimal‐invasive AMIS‐technik zur implantation von hüftprothesen: videobeitrag. Der Orthopäde, 47(9), 782–787. Available from: 10.1007/s00132-018-3591-y 29974162

[6] Grob, K. , Manestar, M. , Ackland, T. , Filgueira, L. & Kuster, M.S. (2015) Potential risk to the superior gluteal nerve during the anterior approach to the hip joint: an anatomical study. The Journal of Bone and Joint Surgery‐American Volume, 97(17), 1426–1431. Available from: 10.2106/JBJS.O.00146 26333738 PMC7535096

[7] Harper, K.D. , Nzeogu, M.I. , Vakil, J.J. , Abdelfadeel, W.M. , Saxena, A. & Star, A.M. (2022) A consistent anatomic landmark for identifying the lateral femoral circumflex artery in a direct anterior hip approach. Orthopedics, 45(5), 262–268. Available from: 10.3928/01477447-20220608-02 35700431

[8] Heinz, T. , Vasilev, H. , Anderson, P.M. , Stratos, I. , Jakuscheit, A. , Horas, K. et al. (2023) The direct anterior approach (DAA) as a standard approach for total hip arthroplasty (THA) in coxa profunda and protrusio acetabuli? A radiographic analysis of 188 cases. Journal of Clinical Medicine, 12(12), 3941. Available from: 10.3390/jcm12123941 37373635 PMC10299585

[9] Huang, X. , Liu, D. , Jia, B. & Xu, Y. (2021) Comparisons between direct anterior approach and lateral approach for primary total hip arthroplasty in postoperative orthopaedic complications: a systematic review and meta‐analysis. Orthopaedic Surgery, 13(6), 1707–1720. Available from: 10.1111/os.13101 34351056 PMC8523754

[10] Ishii, S. , Naito, M. , Kinoshita, K. , Ishimatsu, T. , Akiho, S. & Yamamoto, T. (2019) Effects of lateral circumflex femoral artery ligation on blood flow to the surrounding muscles in the direct anterior approach. HIP International, 29(4), 412–417. Available from: 10.1177/1120700019827487 30729802

[11] Kalhor, M. , Gharehdaghi, J. , Leunig, M. & Ganz, R. (2023) Lateral femoral circumflex artery contribution to the articular and periarticular hip circulation: relevance to the anterior hip approach—a cadaveric study. European Journal of Orthopaedic Surgery & Traumatology: Orthopedie Traumatologie, 33(5), 1547–1555. Available from: 10.1007/s00590-022-03310-2 35727417

[12] Kwak, S.G. & Kim, J.H. (2017) Central limit theorem: the cornerstone of modern statistics. Korean Journal of Anesthesiology, 70(2), 144–156. Available from: 10.4097/kjae.2017.70.2.144 28367284 PMC5370305

[13] Laude, F. (2006) Total hip arthroplasty through an anterior Hueter minimally invasive approach. Interactive Surgery, 1(1), 5Available from: 10.1007/s11610-006-0011-5

[14] Laude, F. , Sariali, E. & Nogier, A. (2009) Femoroacetabular impingement treatment using arthroscopy and anterior approach. Clinical Orthopaedics & Related Research, 467(3), 747–752. Available from: 10.1007/s11999-008-0656-y 19089524 PMC2635440

[15] Leunig, M. , Faas, M. , von Knoch, F. & Naal, F.D. (2013) Skin crease ‘bikini’ incision for anterior approach total hip arthroplasty: surgical technique and preliminary results. Clinical Orthopaedics & Related Research, 471(7), 2245–2252. Available from: 10.1007/s11999-013-2806-0 23412730 PMC3676627

[16] Leunig, M. , Hutmacher, J.E. , Ricciardi, B.F. , Impellizzeri, F.M. , Rüdiger, H.A. & NaalFD, F.D. (2018) Skin crease ‘bikini’ incision for the direct anterior approach in total hip arthroplasty: a two‐ to four‐year comparative study in 964 patients. The Bone & Joint Journal, 100–B(7), 853–861. Available from: 10.1302/0301-620X.100B7.BJJ-2017-1200.R2 29954218

[17] Lüdemann, M. , Kreutner, J. , Haddad, D. , Kenn, W. , Rudert, M. & Nöth, U. (2012) MRT‐basierte messung des muskelschadens nach minimal‐invasiver Hüftprothesenimplantation. Der Orthopäde, 41(5), 346–353. Available from: 10.1007/s00132-011-1889-0 22552541

[18] Meermans, G. , Konan, S. , Das, R. , Volpin, A. & Haddad, F.S. (2017) The direct anterior approach in total hip arthroplasty: a systematic review of the literature. The Bone & Joint Journal, 99–B(6), 732–740. Available from: 10.1302/0301-620X.99B6.38053 28566391

[19] Müller, M. , Tohtz, S. , Dewey, M. , Springer, I. & Perka, C. (2010) Evidence of reduced muscle trauma through a minimally invasive anterolateral approach by means of MRI. Clinical Orthopaedics & Related Research, 468(12), 3192–3200. Available from: 10.1007/s11999-010-1378-5 20458641 PMC2974868

[20] Müller, M. , Tohtz, S. , Dewey, M. , Springer, I. & Perka, C. (2011) Muskeltrauma in der primären Hüftendoprothetik unter Berücksichtigung von Alter und BMI sowie in Abhängigkeit vom operativen Zugangsweg: Minimalinvasiver anterolateraler vs. modifizierter transglutealer Zugang. Der Orthopäde, 40(3), 217–223. Available from: 10.1007/s00132-010-1730-1 21258926

[21] Najima, H. , Gagey, O. , Cottias, P. & Huten, D. (1998) Blood supply of the greater trochanter after trochanterotomy. Clinical Orthopaedics and Related Research, 349, 235–241. Available from: 10.1097/00003086-199804000-00029 9584388

[22] Rachbauer, F. , Kain, M.S.H. & Leunig, M. (2009) The history of the anterior approach to the hip. Orthopedic Clinics of North America, 40(3), 311–320. Available from: 10.1016/j.ocl.2009.02.007 19576398

[23] Spanyer, J.M. , Beaumont, C.M. & Yerasimides, J.G. (2017) The extended direct anterior approach for column augmentation in the deficient pelvis: a novel surgical technique, and case series report. The Journal of Arthroplasty, 32(2), 515–519. Available from: 10.1016/j.arth.2016.08.012 27639306

[24] Takada, R. , Jinno, T. , Miyatake, K. , Hirao, M. , Kimura, A. , Koga, D. et al. (2018) Direct anterior versus anterolateral approach in one‐stage supine total hip arthroplasty. Focused on nerve injury: a prospective, randomized, controlled trial. Journal of Orthopaedic Science, 23(5), 783–787. Available from: 10.1016/j.jos.2018.05.005 29935972

[25] Totlis, T. , Paparoidamis, G. , Terzidis, I. , Piagkou, M. , Tsiridis, E. & Natsis, K. (2020) Surgical anatomy of the lateral circumflex femoral artery branches: contribution to the blood loss control during hip arthroplasty. Annals of Anatomy—Anatomischer Anzeiger, 232. 151566. Available from: 10.1016/j.aanat.2020.151566. 32603828

[26] Zhao, G. , Zhu, R. , Jiang, S. , Xu, N. , Bao, H. & Wang, Y. (2020) Using the anterior capsule of the hip joint to protect the tensor fascia lata muscle during direct anterior total hip arthroplasty: a randomized prospective trial. BMC Musculoskeletal Disorders, 21(1), 21. Available from: 10.1186/s12891-019-3035-9 31926554 PMC6955089

[27] Zhao, G.‐Y. , Wang, Y.‐J. , Xu, N.‐W. & Liu, F. (2019) Dissection and ligation of the lateral circumflex femoral artery is not necessary when using the direct anterior approach for total hip arthroplasty. World Journal of Clinical Cases, 7(24), 4226–4233. Available from: 10.12998/wjcc.v7.i24.4226 31911903 PMC6940332

