# Supplementary figures and images for: High preservation rates of the ascending branch of the lateral femoral circumflex artery during total hip arthroplasty through the direct anterior approach
Source: J Exp Orthop. 2024 Oct 30;11(4):e70066. doi: 10.1002/jeo2.70066 (PMC11522915; doi:10.1002/jeo2.70066)

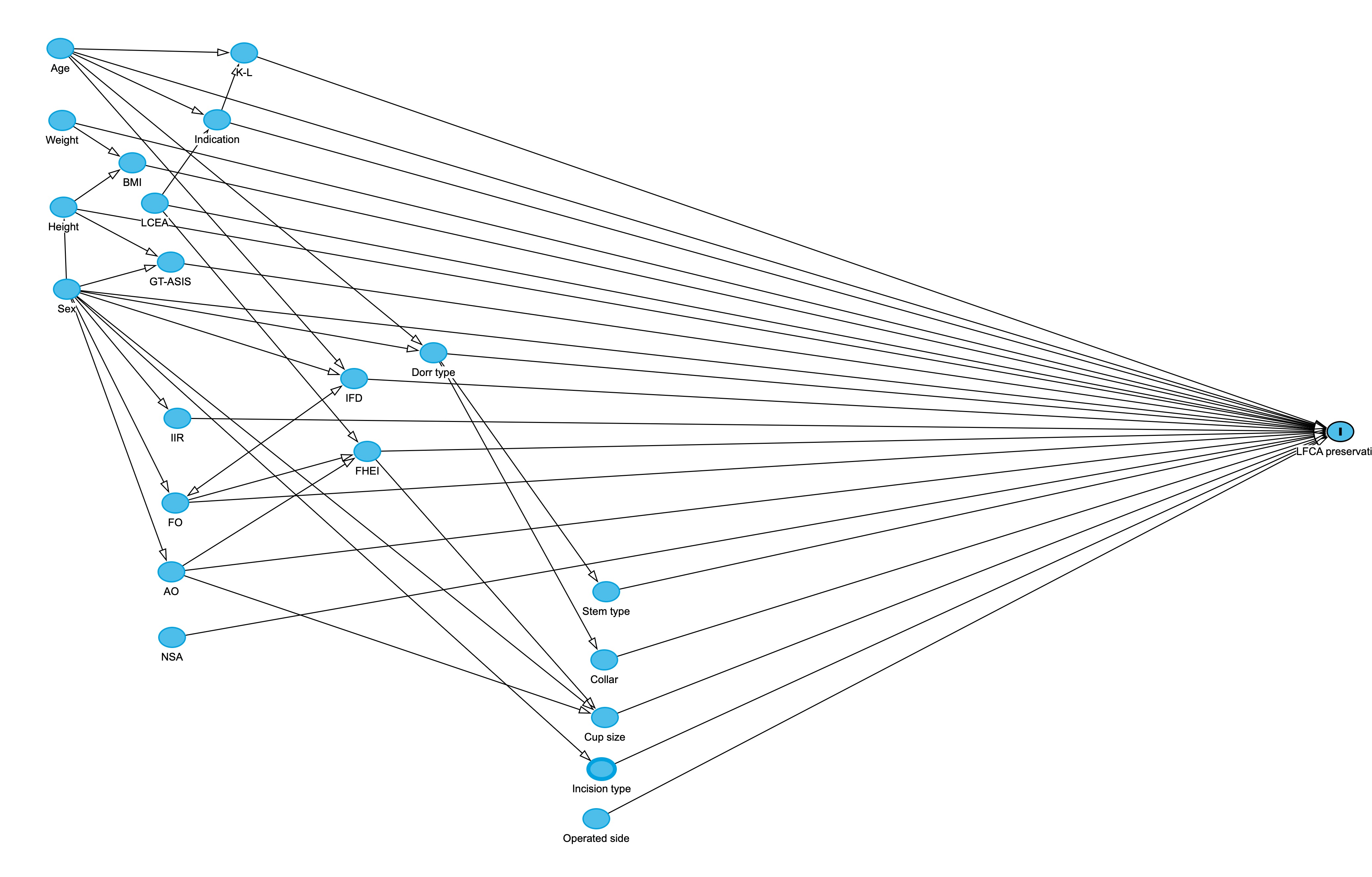

Supplement: Supplementary file 1 — Supporting Information. [file JEO2-11-e70066-s001.jpeg]
